# The role of large language models in improving the readability of orthopaedic spine patient educational material

**DOI:** 10.1186/s13018-025-05955-1

**Published:** 2025-05-28

**Authors:** Melissa Romoff, Madison Brunette, Melanie K. Peterson, Sohaib Z. Hashmi, Michael S. Kim

**Affiliations:** Department of Orthopaedic Surgery, University of California, Irvine, School of Medicine, 101 The City Dr S, Pavilion 3, Building 29 A, Orange, CA 92868 USA

## Abstract

**Introduction:**

Patient education is crucial for informed decision-making. Current educational materials are often written at a higher grade level than the American Medical Association (AMA)-recommended sixth-grade level. Few studies have assessed the readability of orthopaedic materials such as American Academy of Orthopaedic Surgeons (AAOS) OrthoInfo articles, and no studies have suggested efficient methods to improve readability. This study assessed the readability of OrthoInfo spine articles and investigated the ability of large language models (LLMs) to improve readability.

**Methods:**

A cross-sectional study analyzed 19 OrthoInfo articles using validated readability metrics (Flesch-Kincaid Grade Level and Reading Ease). Articles were simplified iteratively in three steps using ChatGPT, Gemini, and CoPilot. LLMs were prompted to summarize text, followed by two clarification prompts simulating patient inquiries. Word count, readability, and accuracy were assessed at each step. Accuracy was rated by two independent reviewers using a three-point scale (3 = fully accurate, 2 = minor inaccuracies, 1 = major inaccuracies). Statistical analysis included one-way and two-way ANOVA, followed by Tukey post-hoc tests for pairwise comparisons.

**Results:**

Baseline readability exceeded AMA recommendations, with a mean Flesch-Kincaid Grade Level of 9.5 and a Reading Ease score of 51.1. LLM summaries provided statistically significant improvement in readability, with the greatest improvements in the first iteration. All three LLMs performed similarly, though ChatGPT achieved statistically significant improvements in Reading Ease scores. Gemini incorporated appropriate disclaimers most consistently. Accuracy remained stable throughout, with no evidence of hallucination or compromise in content quality or medical relevance.

**Discussion:**

LLMs effectively simplify orthopaedic educational content by reducing grade levels, enhancing readability, and maintaining acceptable accuracy. Readability improvements were most significant in initial simplification steps, with all models performing consistently. These findings support the integration of LLMs into patient education workflows, offering a scalable strategy to improve health literacy, enhance patient comprehension, and promote more equitable access to medical information across diverse populations.

## Introduction

Effective education is essential for supporting informed decision-making and protecting patient autonomy [1–5]. However, many educational materials remain inaccessible to the general population due to readability challenges [6]. The National Assessment of Adult Literacy reports that the average U.S. adult reads at an eighth-grade level [7], while Medicaid enrollees average at a fifth-grade level [8]. To address this discrepancy, the American Medical Association (AMA) recommends that patient educational materials be written at or below a sixth-grade reading level to ensure accessibility [9].

Despite these guidelines, a 2018 systematic review found that many health education resources are written at a 10 th- to 15 th-grade level [10]. In the field of orthopaedics, the American Academy of Orthopaedic Surgeons (AAOS) provides public-facing educational content through its OrthoInfo website. Previous studies have demonstrated that orthopaedic educational materials frequently exceed recommended reading levels [11–19], but OrthoInfo’s spine content, despite its complexity and clinical importance, has not been systematically evaluated.

Recent work in other medical specialties has shown that large language models (LLMs) can improve the readability of patient education without compromising accuracy [20–22]. However, adoption in clinical settings remains limited due to concerns surrounding factual reliability, medicolegal implications, and the absence of formal guidelines or validated implementation strategies [23]. These limitations underscore the need for additional research evaluating LLM performance across diverse clinical domains.

Although LLM-based readability analyses have been conducted in orthopaedics, prior studies have not specifically examined spine-related articles from OrthoInfo, a widely used, patient-facing educational platform. Given the procedural complexity and specialized terminology common in spine surgery, general findings from other fields – or even from broader orthopaedic topics – may not fully apply. In this study, we applied a structured, iterative prompting protocol across multiple LLMs to simplify OrthoInfo spine content, evaluating readability improvements, content accuracy, and the potential for effective integration of LLMs into orthopaedic patient education.

## Methods

A cross-sectional observational study of public-facing educational material published by the American Academy of Orthopaedic Surgeons on OrthoInfo.AAOS.org was conducted. Institutional Review Board approval was not required as no protected health information was involved in this study. No large language models (LLMs) were used in drafting this manuscript text itself; however, LLMs were employed for content transformation as part of the study design.

All OrthoInfo web pages associated with the treatment of spinal conditions were identified by selecting “Treatment” from the homepage toolbar and reviewing articles under “Neck” and “Back.” Web pages presenting video content without text substantial enough to enable analysis were excluded. Articles were classified into three categories: 1) Background: Articles primarily providing general education about conditions; 2) Procedure: Articles primarily focused on surgical or non-surgical interventions; 3) Opinion: Articles offering physician perspectives or decision-making advice. Content from each article was extracted as plain text. Words and figures were counted. Date of last review was recorded.

Text was then submitted to the three most used LLMs: ChatGPT GPT-4o (OpenAI, San Francisco, CA), Copilot (Microsoft, Redmond, WA), and Gemini 1.5 Flash-8B (Alphabet, Mountain View, CA), preceded by the request “Please summarize the following.” A new chat session was opened for each article to prevent response bias. Summarization was prompted through three iterative steps: (1) “Please summarize the following,” (2) “I don’t understand, please clarify,” and (3) “I still don’t understand, please clarify.” Each response was documented and analyzed for word count, readability, accuracy, and the presence of an appropriate disclaimer.

Readability was evaluated using Readable (Added Bytes, Hassocks, UK), a software that calculates validated measures of readability. These included the Flesch-Kincaid Grade Level (estimated U.S. school grade level) and Flesch Reading Ease (scored from 0 to 100, with higher scores indicating easier readability). Given the inherent variability of measures of readability, an overall mean readability was calculated by averaging readability measures that provide estimated grade level as had been done in a previous study to allow direct comparison.

Accuracy was independently assessed by two physician authors using a three-point scale: summaries that were fully accurate without requiring correction received three points; those with minor inaccuracies or hallucinations received two points; and those with substantial inaccuracies or hallucinations that affected medical relevance received one point. A three-point scale was selected to facilitate consistent reviewer grading and focus on clinically meaningful content accuracy rather than stylistic variation. In cases of disagreement, scores were reviewed collaboratively, and a consensus was reached between the two reviewers and senior author to ensure consistency and minimize individual bias in the final dataset.

Statistical analysis was performed using R. Descriptive statistics, mean and standard deviations, were calculated for article characteristics and LLM outputs. One-way and two-way ANOVA were used to compare readability, word count, and accuracy across LLMs and steps. Two-way ANOVA was used specifically to evaluate interaction effects between model and simplification step. Assumptions of normality and homogeneity of variants were assessed prior to performing ANOVA tests. Significant ANOVA results were followed by Tukey’s post-hoc tests for pairwise comparisons. Linear regression evaluated trends between readability metrics and baseline article characteristics. Statistical significance was defined as p < 0.05, and visualizations were generated using GraphPad Prism (GraphPad Software, San Diego, CA).

## Results

### Baseline characteristics

Out of the 23 webpages initially reviewed, 19 met the inclusion criteria (8 procedure, 10 background, and 1 ortho-opinion), while 4 were excluded due to being video-based content with minimal written material. No hallucinations were observed during the analysis.

**Fig. 1:**
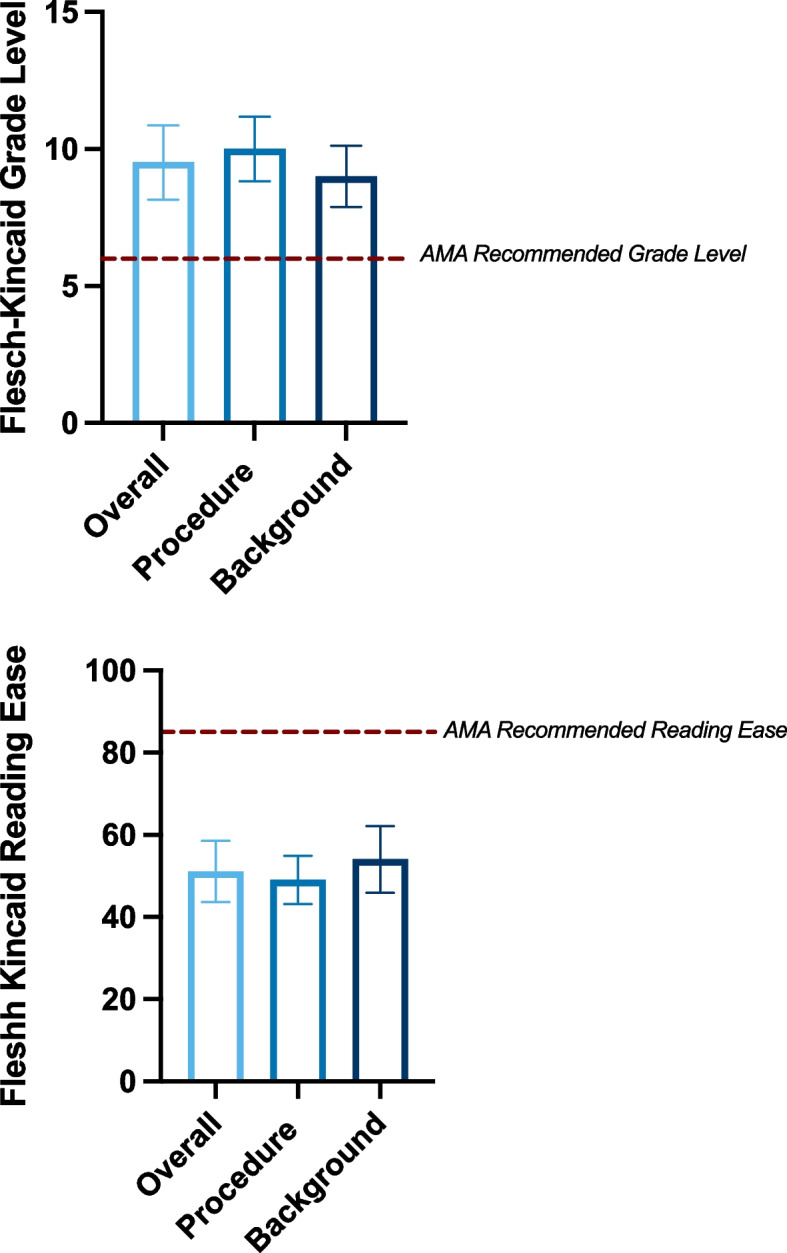
Baseline readability characteristics of OrthoInfo educational materials. Readability metrics are shown for overall (*n* = 19), procedure (*n* = 8), and background (*n* = 10) sections. AMA recommendations are indicated by the red dashed line, and error bars represent standard deviations. **A** Mean Flesch-Kincaid Grade Level for overall, procedure, and background sections. **B** Mean Flesch Reading Ease scores for overall, procedure, and background sections

**Fig. 2:**
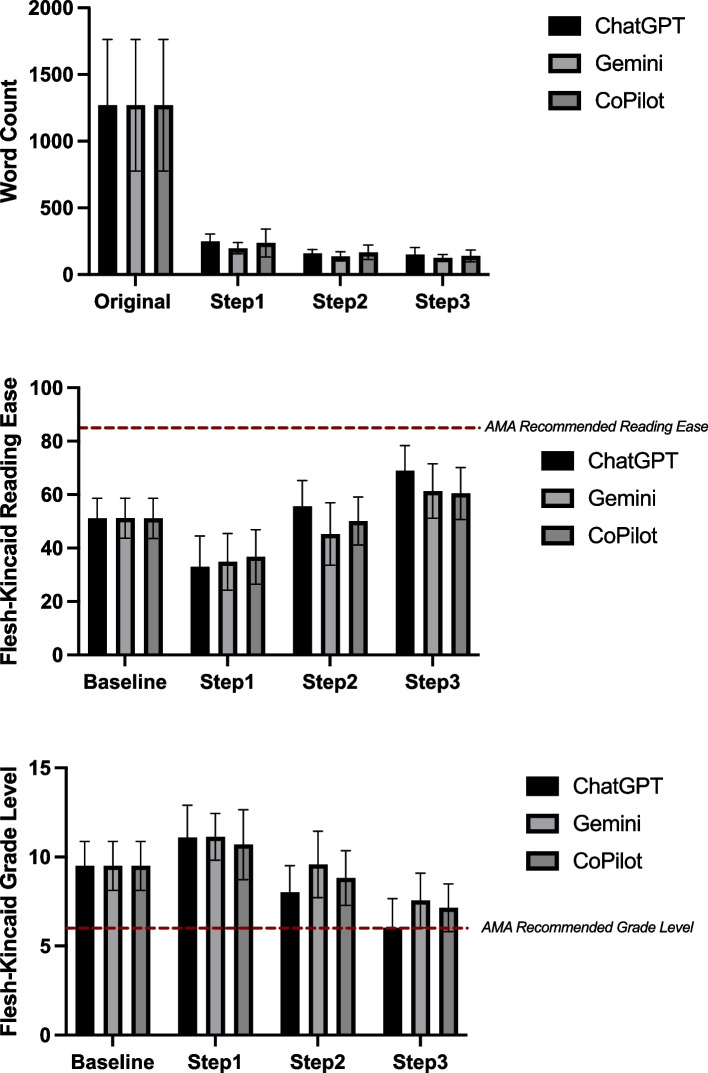
Readability and word count improvements across steps for ChatGPT, Gemini, and CoPilot. Mean values are presented for Baseline (Original) and Steps 1–3, with error bars representing standard deviations. **A** Mean Word Count for ChatGPT, Gemini, and CoPilot across steps. **B** Mean Flesch-Kincaid Reading Ease scores for ChatGPT, Gemini, and CoPilot across steps. **C** Mean Flesch-Kincaid Grade Level for ChatGPT, Gemini, and CoPilot across steps

The baseline readability analysis of the 19 included articles revealed a mean Flesch-Kincaid Grade Level of 9.5 (SD: 1.4), exceeding the AMA’s recommended sixth-grade level, and a mean Flesch Reading Ease score of 51.1 (SD: 7.5), indicating “fairly difficult” readability (Table 1, Fig. 1). On average, the articles contained 1,269 words and included 2.8 figures (Table 1). Procedure articles were shorter, averaging 1,033 words, but were more difficult to read (Grade Level: 9.8; Reading Ease: 49) and contained more figures. (4 per article) (Fig. 1). In contrast, Background articles were longer, averaging 1,523 words, slightly easier to read (Grade Level: 8.7; Reading Ease: 54), and included fewer figures. (2 per article) (Fig. 1).

**Table 1:** Baseline Descriptive Statistics for Readability Metrics

Metric	Overall (Mean ± SD)	Procedure (Mean ± SD)	Background (Mean ± SD)
Word Count	1269 ± 493.86	1033 ± 348.24	1523 ± 474.56
	2.79 ± 2.49	4.0 ± 1.28	2.0 ± 1.02
Flesch-Kincaid Grade	9.52 ± 1.36	9.8 ± 1.18	8.65 ± 1.12
Flesch Reading Ease	51.07 ± 7.48	49.0 ± 5.85	54.0 ± 8.11

### Statistical tests

T-tests revealed that Procedure articles had significantly fewer words than Background articles (*p* = 0.03), but no significant differences were found in figures, readability scores, or grade levels. Pearson correlation analyses showed no significant relationships between readability metrics (Flesch-Kincaid Grade Level and Flesch Reading Ease) and variables such as word count, number of figures, or review age (all *p* > 0.05).

### LLM analysis

**Fig. 3:**
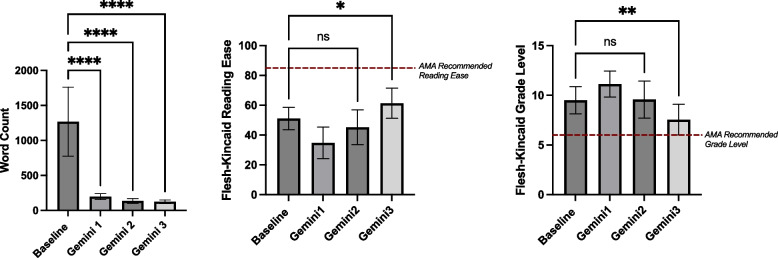
Performance of Gemini in improving readability and reducing word count across steps. Mean and standard deviations for readability metrics (Word Count, Flesch-Kincaid Reading Ease, and Flesch-Kincaid Grade Level) of Baseline articles and across Steps 1–3 using Gemini. Significance levels are indicated within the figure. Error bars represent standard deviations. **A** Word Count for Baseline and Gemini Steps 1–3. **B** Flesch-Kincaid Reading Ease scores for Baseline and Gemini Steps 1–3, with the AMA-recommended reading ease threshold shown as a red dashed line. **C** Flesch-Kincaid Grade Level for Baseline and Gemini Steps 1–3, with the AMA’s recommended sixth-grade reading level shown as a red dashed line

**Fig. 4:**
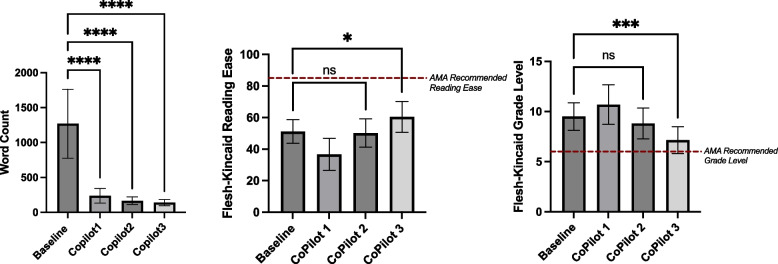
Performance of CoPilot in improving readability and reducing word count across steps. Mean and standard deviations for readability metrics (Word Count, Flesch-Kincaid Reading Ease, and Flesch-Kincaid Grade Level) of Baseline articles and across Steps 1–3 using CoPilot. Significance levels are indicated within the figure. Error bars represent standard deviations. **A** Word Count for Baseline and CoPilot Steps 1–3. **B** Flesch-Kincaid Reading Ease scores for Baseline and CoPilot Steps 1–3, with the AMA-recommended reading ease threshold shown as a red dashed line. **C** Flesch-Kincaid Grade Level for Baseline and CoPilot Steps 1–3, with the AMA’s recommended sixth-grade reading level shown as a red dashed line

**Fig. 5:**
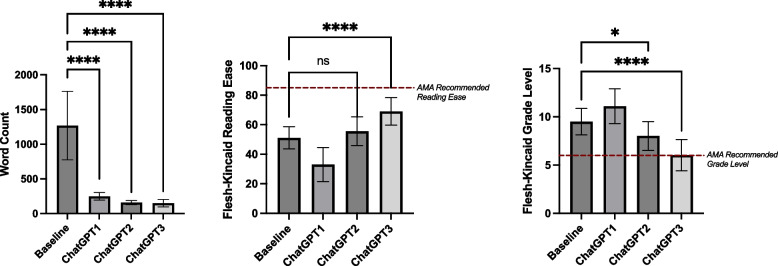
Performance of ChatGPT in improving readability and reducing word count across steps. Mean and standard deviations for readability metrics (Word Count, Flesch-Kincaid Reading Ease, and Flesch-Kincaid Grade Level) of Baseline articles and across Steps 1–3 using ChatGPT. Significance levels are indicated within the figure. Error bars represent standard deviations. **A** Word Count for Baseline and ChatGPT Steps 1–3. **B** Flesch-Kincaid Reading Ease scores for Baseline and ChatGPT Steps 1–3, with the AMA-recommended reading ease threshold shown as a red dashed line. **C** Flesch-Kincaid Grade Level for Baseline and ChatGPT Steps 1–3, with the AMA’s recommended sixth-grade reading level shown as a red dashed line

Each LLM (ChatGPT, Gemini, CoPilot) was evaluated independently across simplification steps. Significant word count reductions were observed for all models (*p* < 0.0001), with the largest reduction occurring between the baseline and Step 1, followed by a plateau in subsequent steps (Table 2, Fig. 2A). Reading Ease improved significantly across steps (*p* < 0.0001), with the greatest improvement between the original articles and Step 1, and smaller but still significant gains from Step 1 to Step 2 (Table 3, Fig. 2B). Grade Level changes were significant (*p* < 0.0001) across steps, but an unexpected initial increase was observed between the original articles and Step 1, with a negative mean difference indicating movement away from the AMA’s sixth-grade recommendation. Subsequent steps showed gradual reductions, ultimately trending toward the desired grade level (Table 3, Figs. 2C, 3, 4 and 5).

**Table 2:** ANOVA Results for Readability Metrics Across ChatGPT, Gemini, and CoPilot

Metric	LLM	F-Statistic	*P*-value	Significant?
Word Count	ChatGPT	89.54	< 0.0001	****
	Gemini	95.87	< 0.0001	****
	CoPilot	86.89	< 0.0001	****
Grade Level	ChatGPT	36.26	< 0.0001	****
	Gemini	17.23	< 0.0001	****
	CoPilot	35.26	< 0.0001	****
Reading Ease	ChatGPT	45.38	< 0.0001	****
	Gemini	23.03	< 0.0001	****
	CoPilot	26.84	< 0.0001	****

**Table 3:** Post-Hoc Results for One-way ANOVA Readability Metrics Across ChatGPT, Gemini, and CoPilot

Metric	Comparison	ChatGPT: Mean Difference (*P*-value)	Gemini: Mean Difference (*P*-value)	CoPilot: Mean Difference (*P*-value)
Word Count	Baseline vs. Step 1	1019 (**** < 0.0001)	1071 (**** < 0.0001)	1031 (**** < 0.0001)
	Baseline vs. Step 2	1110 (**** < 0.0001)	1133 (**** < 0.0001)	1102 (**** < 0.0001)
	Baseline vs. Step 3	1119 (**** < 0.0001)	1114 (**** < 0.0001)	1129 (**** < 0.0001)
Grade Level	Baseline vs. Step 1	−1.595 (* 0.0140)	−1.626 (** 0.0089)	−1.195 (ns 0.0977)
	Baseline vs. Step 2	1.484 (* 0.0255)	−0.07895 (ns 0.9986)	0.6842 (ns 0.5394)
	Baseline vs. Step 3	3.463 (**** < 0.001)	1.942 (** 0.0012)	2.347 (*** 0.0001)
Reading Ease	Baseline vs. Step 1	18.05 (**** < 0.0001)	16.34 (**** < 0.0001)	14.41 (**** < 0.0001)
	Baseline vs. Step 2	−4.474 (ns 0.4819)	5.889 (ns 0.2836)	0.9579 (ns 0.9883)
	Baseline vs. Step 3	−17.90 (**** < 0.0001)	−10.19 (* 0.0141)	−9.321 (* 0.0128)

All LLMs performed similarly in reducing word count, improving reading ease, and lowering grade levels, with no statistically significant differences observed between models or article types. However, a two-way ANOVA revealed that Gemini provided significantly more disclaimers than ChatGPT (mean difference = 10.0, *p* = 0.002) and CoPilot (mean difference = 8.3, *p* = 0.005), while no difference was observed between ChatGPT and CoPilot (Table 4). Simplification steps did not influence disclaimer frequency (*p* = 0.71) (Table 4).

**Table 4:** ANOVA and Post-Hoc Results for Disclaimer Frequency

Effect/Comparison	*F*-value	*P*-value	Mean Difference	Adjusted *P *value	Significance
ANOVA: LLM (Row Factor)	40.79	0.0022	N/A	N/A	N/A
ANOVA: Step (Column Factor)	0.368	0.7131	N/A	N/A	N/A
ChatGPT vs Gemini	N/A	N/A	−10.00	0.0024	**
Gemini vs CoPilot	N/A	N/A	8.333	0.0048	**
ChatGPT vs CoPilot	N/A	N/A	−1.667	0.4206	No

### LLM specific findings

The two-way ANOVA compared all models (ChatGPT, Gemini, and CoPilot) across steps to assess differences in performance. Significant reductions in Word Count were observed across steps (*p* < 0.0001), with no significant differences between models (*p* = 0.80) or interaction effects (*p* = 0.997) (Supplementary Table 1). Reading Ease improved significantly across steps (*p* < 0.0001), with minor differences between models (*p* = 0.03) and a significant interaction effect (*p* = 0.03) (Supplementary Table 1); post-hoc tests indicated ChatGPT slightly outperformed Gemini (mean difference = 4.1, *p* < 0.05), while CoPilot performed similarly to both (Supplementary Table 2). Grade Level reductions were driven by steps (*p* < 0.0001), with small differences between models (*p* = 0.01) but no significant interaction effects (*p* = 0.11) (Supplementary Table 1); Post-hoc tests revealed that ChatGPT achieved slightly lower grade levels than Gemini (mean difference = −0.8, *p* < 0.05), with no significant differences involving CoPilot (Supplementary Table 2). Accuracy varied slightly across steps (*p* = 0.03) but showed no significant differences between models (*p* = 0.95) or interaction effects (*p* = 0.17) (Supplementary Table 1, Supplementary Table 3).

## Discussion

This study evaluated the ability of large language models (LLMs)—ChatGPT, Gemini, and CoPilot—to simplify orthopedic educational content and improve accessibility for patients. The findings demonstrate that LLMs are effective at significantly improving readability metrics such as word count, grade level, and reading ease, with consistent performance across models.

Baseline analysis confirmed that OrthoInfo exceeded the AMA’s recommended sixth-grade reading level, highlighting the need for simplification to make educational materials more accessible. Significant improvements in readability metrics occurred across all models, particularly between the original text and Step 1, with diminishing returns in subsequent steps. While additional iterations may offer further refinement, our data suggest a plateau effect between Steps 2 and 4. We therefore limited the process to three simplifications to balance analytical depth and practical considerations. Future studies could explore extended prompting protocols to determine optimal iteration thresholds.

Interestingly, grade level initially increased after the first simplification step. This may reflect the LLMs’ tendency to introduce polysyllabic or technical language in early summarization attempts, particularly when lacking clear context about the target audience. These changes can inadvertently inflate readability scores, even if the overall content becomes more concise. Subsequent prompts likely encouraged more targeted simplification leading to final outputs that better aligned with the sixth-grade target. Despite, these fluctuations, accuracy remained stable across steps and models, suggesting that LLMS can simplify complex medical content without compromising factual integrity.

Minimal differences were observed between models. ChatGPT slightly outperformed Gemini in both improving reading ease and lowering grade levels, while CoPilot performed similarly to both models. Although some differences were statistically significant, their practical implications appear limited. Importantly, this study was not designed to establish the superiority of any specific LLM, but rather to descriptively assess their current capabilities in simplifying orthopaedic educational content. These findings suggest that the choice of model may be less critical than the simplification process itself.

Several limitations warrant discussion. This study focused exclusively on simplify existing content and did not assess LLMs’ ability to generate new or patient-specific materials. While readability metrics are valuable, they do not fully capture comprehension, particularly for individuals with limited health literacy. Furthermore, although new chat sessions were used for each article to minimize confounding, slight variability in LLM outputs is inherent to generative models. Given our standardized approach and outcome focus, this variability likely had minimal impact.

While this study focused on quantitative outcomes such as readability, word count, and accuracy, it did not include a quantitative or thematic analysis of how content was transformed. Future studies should explore how LLMs affect tone, phrasing and message structure in ways that may influence patient perception. Additionally, while we assessed accuracy using a standardized scale, future studies may benefit from more granular error classification to better understand how LLMs might alter critical content in patient facing materials.

Practical applications of these results involve integrating LLM-based simplification pipelines into electronic health record (EHR) systems to automatically generate accessible versions of home care instructions and procedure notes. Similarly, online educational platforms such as OrthoInfo could integrate LLM-driven readability checks and iterative simplifications prior to publication, ensuring that patient-facing materials meet health literacy standards. Future research should focus on adapting these tools to the needs of specific demographic groups and incorporating direct measures of user comprehension, such as patient surveys or health literacy assessments, to evaluate real-world applicability and maximize impact.

## Conclusion

This study demonstrates the potential of large language models (LLMs) like ChatGPT, Gemini, and CoPilot to improve the readability of orthopedic educational content, addressing the gap between current materials and the AMA’s recommended sixth-grade reading level. By significantly reducing grade levels and improving reading ease, these models simplify medical content without compromising accuracy. While ChatGPT showed slightly greater readability, all models achieved progressive simplifications across steps, highlighting the feasibility of iterative text refinement.

While promising, this study’s focus on simplifying existing materials leaves unexplored opportunities for generating patient-specific content and addressing broader health literacy needs. Importantly, our findings do not assess how LLM-generated content performs across diverse patient demographics or literacy levels. Future research should focus on validating these findings through qualitative studies, including user testing among patients with varying literacy levels and direct patient input to assess comprehension. Systematic efforts are needed to refine LLMs and ensure patient education materials are accessible and effective for all populations.

## Data Availability

No datasets were generated or analysed during the current study.
